# Timing of oral anticoagulant therapy in acute ischemic stroke with atrial fibrillation: study protocol for a registry-based randomised controlled trial

**DOI:** 10.1186/s13063-017-2313-9

**Published:** 2017-12-02

**Authors:** Signild Åsberg, Ziad Hijazi, Bo Norrving, Andreas Terént, Patrik Öhagen, Jonas Oldgren

**Affiliations:** 1 Department of Medical Sciences, Uppsala University, Uppsala, Sweden; 2 Riksstroke, Västerbotten County Council, Umeå, Sweden; 3 Uppsala Clinical Research Center, Uppsala University, Uppsala, Sweden; 4 Department of Neurology, Lund University, Lund, Sweden

**Keywords:** Acute ischemic stroke, Atrial fibrillation, Oral anticoagulation, Randomised clinical trial

## Abstract

**Background:**

Oral anticoagulation therapy is recommended for the prevention of recurrent ischemic stroke in patients with atrial fibrillation (AF). Current guidelines do not provide evidence-based recommendations on optimal time-point to start anticoagulation therapy after an acute ischemic stroke. Non-vitamin K antagonist oral anticoagulants (NOACs) may offer advantages compared to warfarin because of faster and more predictable onset of action and potentially a lower risk of intracerebral haemorrhage also in the acute phase after an ischemic stroke. The TIMING study aims to establish the efficacy and safety of early vs delayed initiation of NOACs in patients with acute ischemic stroke and AF.

**Methods/Design:**

The TIMING study is a national, investigator-led, registry-based, multicentre, open-label, randomised controlled study. The Swedish Stroke Register is used for enrolment, randomisation and follow-up of 3000 patients, who are randomised (1:1) within 72 h from ischemic stroke onset to either early (≤ 4 days) or delayed (≥ 5–10 days) start of NOAC therapy. The primary outcome is the composite of recurrent ischemic stroke, symptomatic intracerebral haemorrhage, or all-cause mortality within 90 days after randomisation. Secondary outcomes include: individual components of the primary outcome at 90 and 365 days; major haemorrhagic events; functional outcome by the modified Rankin Scale at 90 days; and health economics. In an optional biomarker sub-study, blood samples will be collected after randomisation from approximately half of the patients for central analysis of cardiovascular biomarkers after study completion. The study is funded by the Swedish Medical Research Council. Enrolment of patients started in April 2017.

**Conclusion:**

The TIMING study addresses the ongoing clinical dilemma of when to start NOAC after an acute ischemic stroke in patients with AF. By the inclusion of a randomisation module within the Swedish Stroke Register, the advantages of a prospective randomised study design are combined with the strengths of a national clinical quality register in allowing simplified enrolment and follow-up of study patients. In addition, the register adds the possibility of directly assessing the external validity of the study findings.

**Trial registration:**

ClinicalTrials.gov, NCT02961348. Registered on 8 November 2016.

**Electronic supplementary material:**

The online version of this article (doi:10.1186/s13063-017-2313-9) contains supplementary material, which is available to authorized users.

## Background

In Sweden approximately 20,000 patients are annually hospitalised for a first or recurrent ischemic stroke [1]. Atrial fibrillation (AF), one of the most important risk factors for stroke, is prevalent among these patients, ranging from 10% in those aged < 65 years to 40% in those aged > 85 years [2, 3]. Oral anticoagulation (OAC) therapy is well established and highly recommended for the prevention of recurrent ischemic stroke in patients with AF [4–7]. Non-vitamin K antagonist oral anticoagulants (NOACs) compared to warfarin are, in randomised controlled trials (RCT), demonstrated to be at least as effective for stroke prevention, with equal or lower risk for major bleedings in patients with AF [8]. Subgroup analyses also confirm the results in patients with prior stroke [9–11]. However, the pivotal large-scale studies of NOACs vs warfarin do not address the setting of acute stroke, since patients with a recent stroke (within 7–14 days) were excluded from these studies [12–15]. The prevention of recurrent ischemic strokes by early initiation of NOAC might be offset by an increased risk for haemorrhage, in particular intracerebral haemorrhage (ICH). This concern is supported by a meta-analysis of seven RCTs of non-oral anticoagulation (heparins) started within 48 h vs aspirin or placebo, indicating that very early parenteral anticoagulation was associated with increased symptomatic ICH without significantly reducing recurrent ischaemic stroke, mortality or disability [16]. Conversely, more recent observational studies on OAC therapy in the acute setting indicates possible benefit of an early (< 14 days) initiation [17–20]. A European prospective observational study, including mainly patients treated with warfarin and/or low molecular weight heparin, proposed days 4–14 from stroke onset to be the best time to initiate OAC treatment; however, the authors also emphasised the need of a RCT for assessing the efficacy of NOACs in the acute phase [19]. In a recent Asian prospective observational study, NOACs were initiated five days (median) after stroke onset with no subsequent occurrence of ICH, not even in patients with severe stroke [20].

Due to the sparse evidence, current national and international guidelines do not provide specific recommendations regarding the best time point to start OAC therapy, other as consensus statements [4–7]. For example, the 2014 American guidelines suggest that in most patients with an acute ischemic stroke and AF, it is reasonable to initiate OAC within 14 days [7]. NOAC may offer several potential advantages in the acute setting after ischemic stroke in AF patients; all NOAC have a much faster and more predictable onset of action and, most importantly, a substantial lower risk of ICH compared to warfarin [8, 9]. Despite these advantages, there is an important and urgent unmet need to find the optimal time when to start NOAC therapy as secondary stroke prevention in patients with AF [21].

Therefore, the primary objective of the study is to compare early vs delayed initiation of NOAC for efficacy and safety in patients with acute ischemic stroke and AF. The secondary objectives are to assess the impact of early vs delayed initiation of NOAC on functional outcome, NOAC adherence/persistence and health economy, in addition to the development and identification of prognostic biomarkers in the sub-study.

## Methods

### Design

**Fig. 1:**
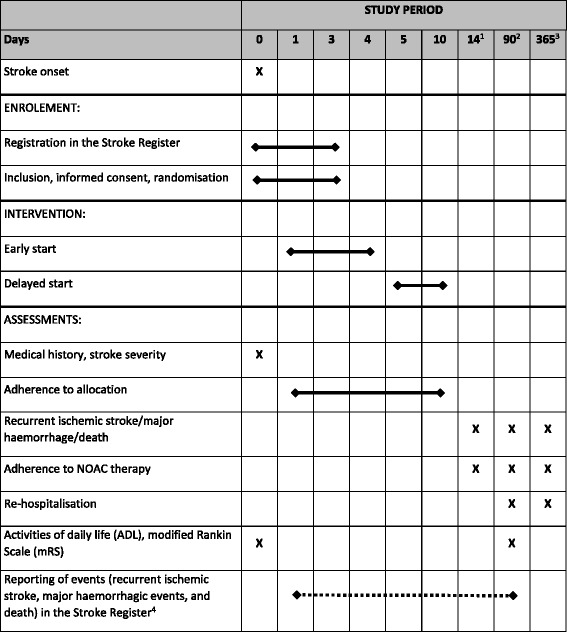
Schedule of enrolment, intervention and assessments

The prospective TIMING study is a national, investigator-led, registry-based, multicentre, open-label, non-inferiority, RCT. The intervention in this study is not the pharmacological agents (i.e. apixaban, dabigatran, edoxaban and rivaroxaban) per se, but rather the actual timing of treatment initiation. This registry-based RCT (R-RCT) will use the Swedish Stroke Register for enrolment, randomisation and follow-up [1]. Additional data will be retrieved through record linkage with mandatory national registers. An overview of the study is presented in Fig. 1 and a SPIRIT Checklist is included as Additional file 1.

### Inclusion and exclusion criteria

The target population is adult patients (aged ≥ 18 years) with acute ischemic stroke associated with AF and an indication for NOAC treatment. AF includes paroxysmal, persistent and permanent AF and can be previously known or diagnosed during index hospitalisation. The study population will consist of patients identified at the Stroke Unit, fulfilling the eligibility criteria, and who give their written informed consent (Table 1). Approximately 40 of the 72 Stroke Units in Sweden will participate in the study. Patients with ongoing NOAC and warfarin therapy at index stroke are considered eligible if NOAC is interrupted and PK-INR is < 1.7, respectively. Participation in the study is voluntary and patients will receive all relevant information on the study treatment and procedures in order to decide whether or not to participate and of the possibility to withdraw from the study at any time-point. All study data will be handled with strict confidentiality.

**Table 1:** TIMING inclusion/exclusion criteria

*Inclusion criteria*	
• Adult patients (aged ≥ 18 years) with acute ischemic stroke and atrial fibrillation	
• Eligible and willing to start (or restart) NOAC	
• Signed informed consent	
*Exclusion criteria*	
• Contraindication to NOAC (e.g. ongoing bleeding, mechanical heart valve prosthesis)	
• Ongoing therapy with NOAC (without ≥ 2 days interruption at index stroke)	
• INR > 1.7	
• No second brain imaging (CT/MRI) after thrombolysis/thrombectomy	
• Previous randomisation in the TIMING study	

*NOAC* non-vitamin K antagonist oral anticoagulant drugs, *INR* international normalised ratio, *CT* computed tomography, *MRI* magnetic resonance imaging

### Randomisation

Eligible patients will be randomised by the treating physician at the patient’s local hospital, within 72 h from stroke onset. The choice of NOAC (i.e. apixaban, dabigatran, edoxaban or rivaroxaban) is at the discretion of the treating physician as all four are available and reimbursed in Sweden. If eligible and willing to participate, the patient will be given a study specific ID and thereafter be randomly allocated, equal in each study arm, to early (≤ 4 days) or delayed (≥ 5–10 days) start of NOAC by a central computer within the Swedish Stroke Register/R-RCT infrastructure.

### Outcomes

Primary composite outcomes:

- Recurrent ischemic stroke within 90 days, defined as a new focal neurological deficit of sudden onset lasting at least 24 h (or < 24 h if following therapeutic intervention, i.e. thrombolysis or thrombectomy, or if the deficit results in death < 24 h), occurring > 24 h after the index ischemic stroke, irrespective of vascular territory and that is not attributable to oedema, brain shift, haemorrhagic transformation, intercurrent illness, hypoxia or drug toxicity [22]; and/or
- Symptomatic ICH within 90 days, defined as a new focal neurological deficit of sudden onset lasting at least 24 h with documented ICH on imaging (CT or MRI). Any intraparenchymal hematoma (≥ 10 mm) will be considered, including haemorrhagic transformation of the index ischemic stroke. However, microhaemorrhages (< 10 mm) do not fulfil the study definition of ICH. ICH will be classified as symptomatic if it is associated with ≥ 4 points increase in total National Institutes of Health stroke scale (NIHSS) or ≥ 2 points increase in 1 of the NIHSS categories [23]; and/or
- Death considered as all-cause mortality within 90 days.

Secondary outcomes

- The individual components of the primary outcome at 90 days and up to 365 days;
- Functional outcome at 90 days as defined by the modified Rankin Scale (mRS);
- Major haemorrhages at 90 days are those that result in death or are life-threatening as defined by the International Society on Thrombosis and Haemostasis (ISTH) [24] or consume major healthcare resources, which include:
  - fatal bleeding; and/or
  - symptomatic bleeding in a critical area or organ, such as intracranial (including asymptomatic ICH), intraspinal, intraocular, retroperitoneal, intra-articular or pericardial, or intramuscular with compartment syndrome; and/or
  - bleeding causing a fall in haemoglobin level of ≥ 20 g/L, or leading to transfusion of two or more units of whole blood or red cells; and/or
  - bleeding events leading to hospitalisation (in addition to the original ISTH definition [24]).
- Adherence/persistence to NOAC therapy at 90 days;
- Days at Acute or Comprehensive Stroke Unit;
- Length of hospital stay within 90 days;
- Re-admission to hospital within 90 days;
- Health economy analysis.

### Data management

All study-specific data, collected in the Swedish Stroke Register by the study nurse or stroke coordinator at each participating hospital, will be automatically transferred to the TIMING study database. Any changes of data in the Swedish Stroke Register are tracked (audit trail) in the study database. To minimise incompleteness and to increase the correctness of data, logical controls for critical data are programmed in the Swedish Stroke Register. Additional data management will be performed at Uppsala Clinical Research Center. After study completion and database lock, a request will be sent to Swedish Board of Health and Welfare for additional data from the mandatory public National Patient, Cause of death, and Prescribed Drug registers to be merged with the TIMING analysis database.

The statistical analyses will be performed by Uppsala Clinical Research Center and the results will be presented to the investigators. Based on these data, the steering committee, in cooperation with the investigators, will prepare a manuscript intended for publication in a medical/scientific journal.

### Biomarker sub-study

Recently developed and validated biomarker-based risk models with established biomarkers for prediction of stroke and severe haemorrhages (ABC-stroke and ABC bleeding risk score) revealed that biomarker-based risk models are superior to the currently used risk models based on clinical variables only [25–27]. However, the biomarker-based ABC risk models have never been assessed in the acute setting following an ischemic stroke. Blood samples will be collected as soon as feasible after randomisation from patients who give their informed consent to participate in the TIMING substudy. In total, two EDTA tubes of 6 mL each will be collected. Analyses will be performed at the Uppsala Clinical Research Center laboratory and include biomarkers, e.g. cardiac troponin, NT-proBNP, and GDF-15. In subsequent steps, exploratory analyses are planned to identify novel pathophysiological and/or prognostic biomarkers in ischemic stroke and AF. Based on prior statistical evaluations, blood samples will be collected from 1500 patients participating in the TIMING study [28].

### Data and safety monitoring board

An independent data and safety monitoring board (DSMB) will monitor patient safety and the study conduct. The DSMB will hold regular meetings to ensure there is no imbalance in endpoints, with focus on recurrent ischemic strokes and intracerebral haemorrhages. In case of revealed harm or overwhelming superiority associated with one of the treatment arms, the DSMB will recommend premature termination of the study.

### Sample size estimates

To address paucity of data on early stroke recurrence rate in Sweden, we performed a retrospective, medical record review study (the Pre-TIMING study) to investigate the stroke recurrence rate in patients with acute ischemic stroke and AF during the index hospitalisation. This observational study included all consecutive patients with acute ischemic stroke and AF registered in The Swedish Stroke Register during 2014 at Skåne University Hospital, Lund and Uppsala University Hospital. The results of this study revealed 4% recurrent ischemic stroke and 3% symptomatic ICH during the index hospitalisation (mean = 16 days) and 4% all-cause mortality at 90 days in patients with OAC therapy [29].

The sample size calculation in TIMING is based on findings from the Pre-TIMING study [29]. The proportion of events of the primary composite variable at 90 days is assumed to be 12% in both treatment regimes. The non-inferiority margin is set to be 0.03. The selection of non-inferiority margin is based on magnitudes of commonly observed and meaningful relative and absolute risk reductions in a wide range of secondary stroke preventive trials [30]. Based on these assumptions, a sample size of 1451 patients per group would be sufficient to test the primary hypothesis (non-inferiority) with a power of 80% using a significance level of 5%. Although there is no loss to follow-up in the National Board of Health registers, we conservatively set the sample size to 3000 patients.

### Statistical analyses

The primary composite outcome of new ischemic stroke or symptomatic ICH or all-cause mortality within 90 days will be analysed using an ordinary z-test for proportions. The hypothesis to be tested is if early start of NOAC treatment is non-inferior to delayed start of treatment. Non-inferiority will be tested by comparing the lower limit of the two-sided confidence limit for the difference in proportions of events for the two treatment regimens to the suggested delta value (i.e. the non-inferiority margin). If the data support the non-inferiority claim, the hypothesis of superiority will also be tested; a testing strategy supported by current guidelines [31]. A Cox proportional hazard model will be used as a sensitivity analysis. Differences between treatment regimens for the individual components of the primary composite variable will be tested as secondary outcomes. Subgroup analyses will be performed based on age, sex and stroke severity.

The Modified intention-to-treat population is the primary efficacy and safety dataset and will consist of all randomised individuals who receive at least one dose of NOAC treatment during the treatment period (Modified intention-to-treat population). Participants will be presented in the treatment start group to which they were randomised (even if the treatment started at a time point different than what was decided by the randomisation). The Per protocol population will be a subset of the Modified intention-to-treat population. All data points after a relevant protocol deviation will be excluded from this dataset. This dataset will be used for sensitivity analyses of the primary efficacy endpoint if > 10% of participants in any treatment group have relevant protocol deviations.

## Discussion

The TIMING study addresses the hitherto unmet need to find the optimal timing to start NOAC therapy as secondary prevention in patients with acute ischemic stroke and AF. This clinical dilemma is common [1–3], and studies similar to TIMING are planned both in Europe and US (clinicaltrials.gov identifiers NCT03148457; NCT03021928). Due to inadequate scientific evidence in the acute setting, the recommendations given in international guidelines for starting or restarting OAC therapy are based on consensus [4–7]. Thus, the TIMING study has the potential to provide solid evidence to guide treatment decisions in the future. The results of the biomarker sub-study may also expand the knowledge on risks for stroke, haemorrhage and death in this vulnerable population.

RCTs are fundamental to provide clinical evidence to guide physicians in their selection of treatment options. However, RCTs have their own limitations, such as selection bias of trial centres and patients, thus not necessarily representing real world practice. Moreover, the costs of conducting large adequately powered traditional RCTs may be overwhelmingly high to address many clinically relevant issues. By the inclusion of a randomisation module in a large inclusive clinical registry with unselected consecutive enrolment, the advantages of a prospective randomised trial can be combined with the strengths of a large-scale all-comers clinical registry. This concept of a R-RCT has been described as a disruptive technology in clinical research [32] and several large-scale R-RCTs performed in Sweden have recently been published [33–35]. The R-RCT concept may, beyond the simplified enrolment and follow-up of study patients, also facilitate the translation of clinical trial evidence into routine clinical practice. Furthermore, the progress of such practice changes in regular healthcare is easy to identify and follow-up in clinical registries such as the Swedish Stroke register.

### Trial status

The study is ongoing and planned to continue throughout 2019. Enrolment of patients started in April 2017. Protocol version 2.0, 27 February 2017.

## Additional file

Additional file 1: SPIRIT 2013 Checklist: Recommended items to address in a clinical trial protocol and related documents. (PDF 244 kb)

## Abbreviations

ABC-score: Age, biomarkers, clinical history-score
ADL: Activities in daily living
AF: Atrial fibrillation
CT: Computed tomography
DSMB: Data and safety monitoring board
EDTA: Ethylenediaminetetraacetic acid
GDF-15: Growth differentiation factor-15
GI: Gastrointestinal
ICH: Intracerebral haemorrhage
ISTH: International Society of Thrombosis and Haemostasis
MRI: Magnetic resonance imaging
NIHSS: National Institutes of Health stroke scale
NOAC: Non-vitamin K antagonist oral anticoagulant
NT-proBNP: N-terminal pro-B-type natriuretic peptide
OAC: Oral anticoagulant
RCT: Randomised controlled trial
R-RCT: Registry-based randomised clinical trial
